# Effects of noisy galvanic vestibular stimulation on spatial memory in virtual reality

**DOI:** 10.1038/s41598-025-02252-z

**Published:** 2025-07-01

**Authors:** Purav Bhardwaj, Misha Sra

**Affiliations:** Human-AI Experience (HAX) Lab, University of California, Santa Barbara, 93106 USA

**Keywords:** Electrical and electronic engineering, Computer science

## Abstract

Spatial memory and navigation are foundational cognitive functions intricately tied to the hippocampal and striatal neural circuits. These regions integrate multisensory inputs from the environment, with the vestibular system exerting a particularly strong influence on visuospatial processing. While prior work has explored how Galvanic Vestibular Stimulation (GVS) can enhance spatial cognition in individuals with vestibular disorders, limited research has focused on its potentially beneficial effects in those without vestibular disorders. To address this gap, we present a study using a novel experimental paradigm that combines noisy GVS (nGVS) with virtual reality (VR) to systematically examine the impact of vestibular stimulation on spatial learning and memory in healthy adults. Our findings (n=32) suggest that nGVS can significantly improve spatial memory performance, facilitating learning and recollection compared to the without-nGVS condition. Unlike previous screen-based studies, our work uniquely integrates nGVS with an ecologically valid scenario in VR, with study results indicating nGVS as a potential modifier of human spatial memory.

## Introduction

Spatial navigation is an indispensable human activity that necessitates the temporal integration, preservation, and evaluation of multiple different spatial cues. Central to these tasks is spatial memory, the cognitive faculty that archives and recalls spatial data. It allows us to encode, store and retrieve information about the layout of spaces, location of objects within them, and their spatial relationships^1–3^. For example, we can remember the location of specific objects, like the fridge in the kitchen or the couch in the living room. We can also remember the location of specific objects in the fridge and others around the house.

The ability to recall the location of objects is critical for many everyday tasks such as finding items at home or in a store. Age-related cognitive decline can negatively impact spatial memory, making it difficult to encode and retrieve spatial information, like where an object was last seen^4^. Investigating object-location memory is of high relevance as impairments are associated with early symptoms of mild cognitive decline and Alzheimer’s disease^5,6^.

Finding an object in space involves multiple cognitive processes which include knowledge of environmental features, decision-making, path integration, goal and location identification, required to retrieve object locations^7^. To find an object, one needs to remember its location, plan a path to get there, and recognize the object when one arrives. All of these require another set of cognitive processes, including memory, spatial reasoning, and attention. Each of these processes mandates the seamless integration of multiple spatial cues to construct a holistic mental model of the environment. Extrinsic environmental cues, such as salient landmarks or light sources, serve as spatial anchors. This information is represented either in relation to the body (egocentric frame) or in relation to the space and objects (allocentric frame) to guide navigation^8^. However, spatial cognition is not exclusively tethered to these external beacons. Intrinsic cues, predominantly those from the vestibular system allow a person to infer location or orientation relative to a reference^9^. Both intrinsic and extrinsic cues need to be integrated for effective spatial navigation.

The vestibular system has been shown to have a particularly strong influence on visuospatial memory. Animal experiments from as far back as the early 1960s show that altering one or both vestibular labyrinths is associated with failure to remember new spatial locations^10^. This evolutionarily ancient sensory system^11^, often subsumed under proprioception, provides vital information about body position and interrelations in space, playing a crucial role in balance, head-eye coordination, and effective spatial navigation^12^. Vestibular disorders in humans not only result in balance and postural issues but have also been shown to be associated with hippocampal atrophy^13^. In animals studies, vestibular lesions have been shown to directly impact head-direction and place-cell activity leading to impaired spatial memory^14,15^.

Galvanic Vestibular Stimulation (GVS) is a non-invasive technique that has been used in prior work to stimulate the vestibular system^16,17^. It works by delivering weak currents transcutaneously through electrodes that are placed over the mastoid processes behind the ears. Common approaches include applying either constant current or randomly fluctuating currents which is known as Noisy GVS (nGVS). Human neuroimaging studies have shown activation of the hippocampus and the striatal regions with vestibular stimulation^18,19^. Given this and the fact that the vestibular system plays a role in visuospatial learning, it can be expected that activating the vestibular system could modulate human spatial memory.

The galvanic vestibular stimulation (GVS) mechanism modulates neural activity in the vestibular afferents through transcutaneous electrical currents applied over the mastoid processes, behind each ear. Electrophysiological research has demonstrated that both natural and artificial vestibular stimulation modulates hippocampal activity, specifically affecting place cell firing patterns and theta oscillations, highlighting the direct influence of the vestibular system on the hippocampus, and thereby, spatial memory function^20,21^. For motion sickness reduction, GVS appears to work through a separate pathway involving the nucleus tractus solitarius (NTS) and vestibular nuclei, which modulate autonomic responses and suppress conflicting sensory signals that typically trigger symptoms of motion sickness such as dizziness, vertigo and nausea^22^. This autonomic pathway is distinct from the hippocampal circuit involved in spatial memory, though both share initial vestibular nerve activation.

In this work, we present a study to investigate the potential impact of nGVS on spatial memory, using a virtual reality (VR) spatial navigation task (Fig. 6). Traditionally, visuospatial studies in humans have been performed using paper-and-pencil tasks^23,24^ or computer screen-based tasks^25,26^. Often these tasks present spatial cues, even when using 3D, that are radically different from those available during egocentric navigation in the real world. However, real-world navigation tasks are expensive to create and potentially dangerous with limited ability to modify spatial cues and control study variables^27,28^, making it challenging to perform user experiments. VR provides a crucial advantage in spatial memory research by bridging the gap between traditional screen-based methods and real-world environments. Unlike 3D environments displayed on 2D screens, VR offers a first-person, egocentric perspective that closely mimics reality. A user’s sense of “presence” in the designed virtual environment leads to realistic behaviors^29^, which significantly enhances the ecological validity of spatial memory study results.

While nGVS has been shown to have a positive effect on balance^30^ and walking^31^ in humans, only recently did Hilliard et al.^32^ investigate its effect on spatial cognition. The task was presented on the computer screen and participants were shown 4 objects, one at a time, and asked to remember their locations. Since the objects are all placed on an open plane, with mountains in the far distance to provide orientation cues, a location cue in the form of a construction cone is placed on the flat plane to assist user memorability. In contrast to their study environment, we created a VR environment that incorporates object occlusions and lighting conditions that more closely mimic real-world settings, where objects can be behind others and not always clearly visible. We believe this approach allows for a more authentic spatial experience, potentially yielding results that better reflect natural spatial memory processes. Our initial findings suggest that nGVS markedly enhances spatial performance. Participants in the with-nGVS condition consistently demonstrated optimized and efficient navigational trajectories, taking shorter paths and less time to locate the objects than those in the without-nGVS condition. While there is a chance that motion sickness reduction with GVS may have improved spatial memory, prior research points to different brain pathways for spatial memory and motion sickness. GVS modulates hippocampal activity^20,21^, whereas its motion sickness benefits operate through separate autonomic pathways involving the nucleus tractus solitarius^33^, suggesting independent mechanisms despite shared initial vestibular nerve activation.

## Results

It is important to note that our study serves as an initial exploration of this specific VR-based spatial memory task with nGVS. While we perform statistical data analysis, the results need to be interpreted cautiously and not considered broadly generalizable. Instead, these findings should be viewed as valuable indicators and potential directions for future, larger-scale studies in this area of research. Our analysis began with first conducting the Shapiro-Wilk test to assess the normality of our data distribution for the with-nGVS and without-nGVS conditions. The test results confirmed that our data did not follow a normal distribution. Therefore, we chose the Mann-Whitney *U* (MWU) test, a non-parametric method for comparing two independent groups without the presumption of normality. This test was particularly appropriate for our study for several reasons. It is robust to smaller sample sizes, accommodating our study’s scale, and it applies to continuous data, matching our data’s nature. This approach allowed us to explore the differences in spatial memory performance between the with-nGVS and without-nGVS conditions with methodological integrity. Primary performance metrics in our study are path length (PL) and time to completion (TTC).

### Path lengths

**Fig. 1:**
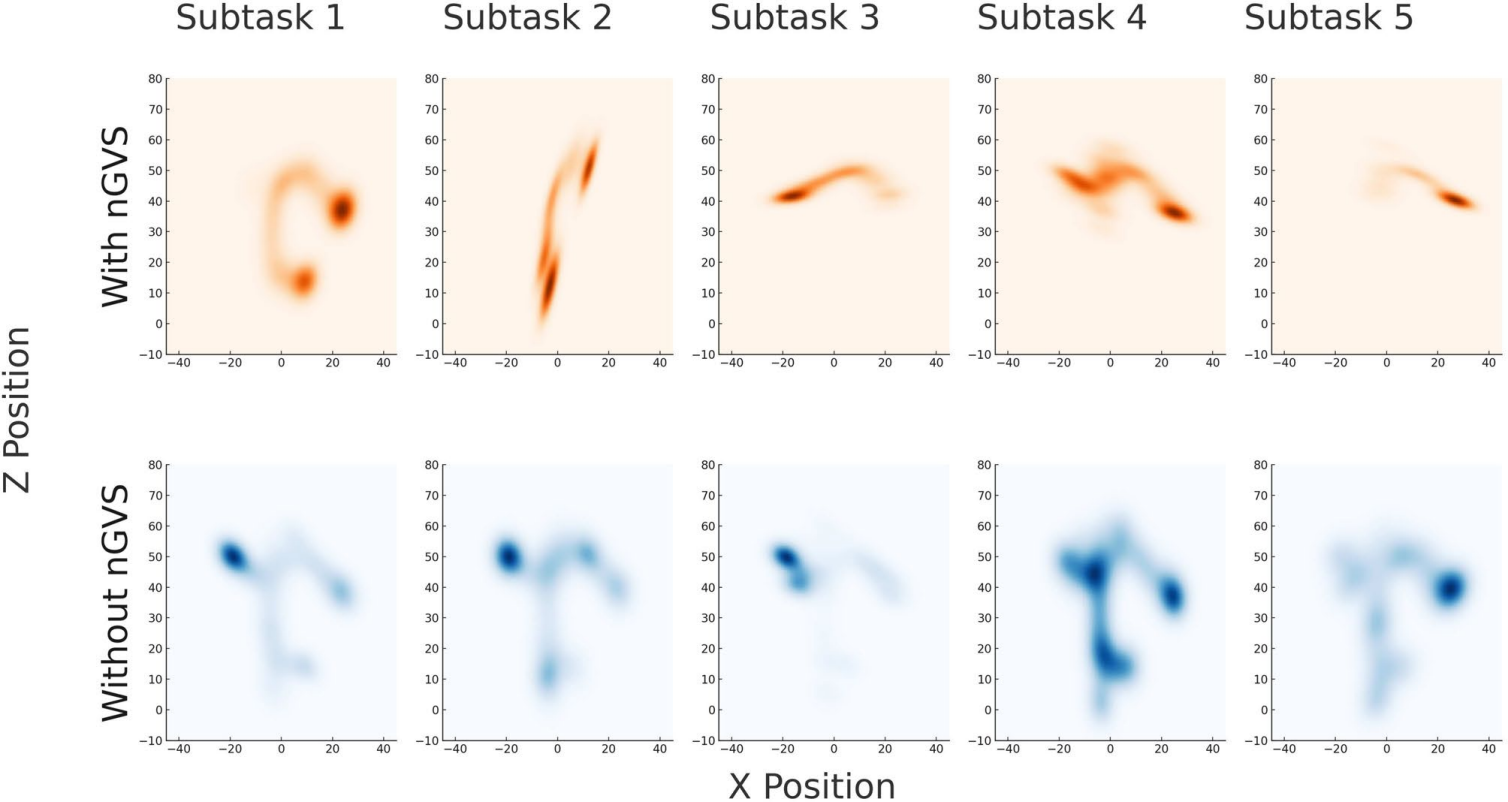
Heatmaps of participant paths plotted in the XZ ground plane (top-down view) for each subtask, for both with-nGVS and without-nGVS conditions. Darker color indicates higher densities of path i.e., participants visited those locations more than others. When comparing the participant path maps across conditions, we observed a notable difference. In the without-nGVS condition, participants’ paths showed more meandering and exploratory behavior. In contrast, the paths in the with-nGVS condition appeared more direct and purposeful. This suggests that the application of nGVS may have influenced participant navigation strategies and spatial awareness.

For each pair of consecutive coordinates in the path trajectory, we computed the Euclidean distance. These coordinates were obtained from Unity, the 3D development platform used for the experiment. The path length was calculated by summing all the computed distances. This sum represents the total length of the path taken by a participant for that sub-task. The non-parametric Mann-Whitney *U* test was employed to compare the distributions of path lengths between the with-nGVS and without-nGVS conditions. Figure 1 shows the heatmaps of paths plotted for each subtask for both with- and without-nGVS conditions. The analysis revealed a statistically significant difference in path lengths between the with-nGVS and without-nGVS conditions ($U = {926}$, $p < 0.0001$), indicating that the median path length in the with-nGVS condition diverges substantially from that in the without-nGVS condition. This finding suggests that the presence of vestibular stimulation in the with-nGVS condition may contribute to enhanced spatial navigation efficiency, as reflected by the reduced path lengths compared to the without-nGVS condition.

**Fig. 2:**
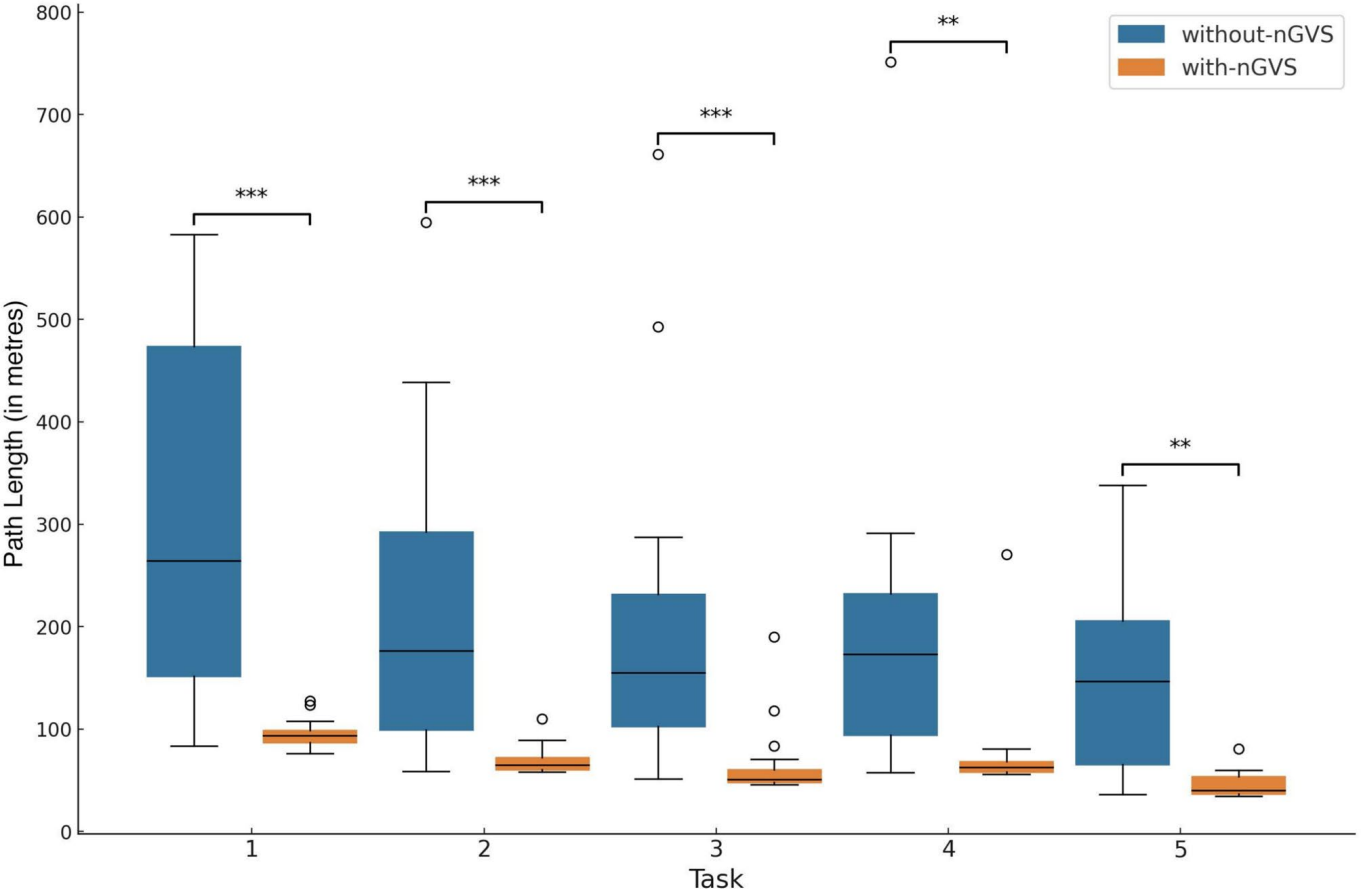
Boxplot comparison of path lengths across subtasks, highlighting the distinction between the with-nGVS and without-nGVS conditions. The plot reveals that paths in the with-nGVS condition are consistently shorter compared to the longer paths observed in the without-nGVS condition.

#### Subtask analysis

A more granular analysis on a subtask basis reveals nuanced differences in navigation performance. The MWU test statistics and corresponding p-values for each task are presented in Table 1, highlighting the variations in navigation performance across the five subtasks. Figure 2 shows a comparison of path lengths across subtasks indicating the differences between the with- and without-nGVS conditions.

**Table 1:** Mann-Whitney U test results and Cliff’s delta by task.

Sub-task	U statistic	*p*-value	Cliff’s delta
1	31.0	$p<$ 0.001	−0.758
2	29.0	$p<$ 0.001	−0.773
3	27.0	$p<$ 0.001	−0.789
4	42.0	$p<$ 0.001	−0.672
5	41.0	$p<$ 0.001	−0.680

All subtasks demonstrated significant differences between conditions ($p<$ 0.001). These results suggest consistent differences in path lengths between the with-nGVS and without-nGVS conditions across all navigation subtasks. Post-hoc analyses using both Bonferroni and Holm-Bonferroni corrections for multiple comparisons confirmed the significance of these differences (all corrected $p<$ 0.007). The most pronounced differences were observed in paths 1–3 (corrected $p<$ 0.001), with paths 4 and 5 showing slightly higher but still highly significant corrected *p*-values ($p<$ 0.007).

#### Effect size consideration

The magnitude of differences observed was quantified using Cliff’s Delta ($\delta$), with values presented in Table 1. These effect sizes substantiate the significant influence of GVS on spatial navigation tasks across all subtasks, where moderate to large deltas were observed. The deltas indicate a substantial effect size, particularly for subtasks 2 and 3 ($\delta = -0.773$, $\delta = -0.789$, respectively), suggesting a stronger effect of nGVS in terms of path length efficiency. In summary, the significant results from the MWU test, coupled with the large effect sizes indicated by Cliff’s Delta, affirm the substantial role of nGVS in enhancing spatial navigation within virtual environments in this study.

### Time to complete task

**Fig. 3:**
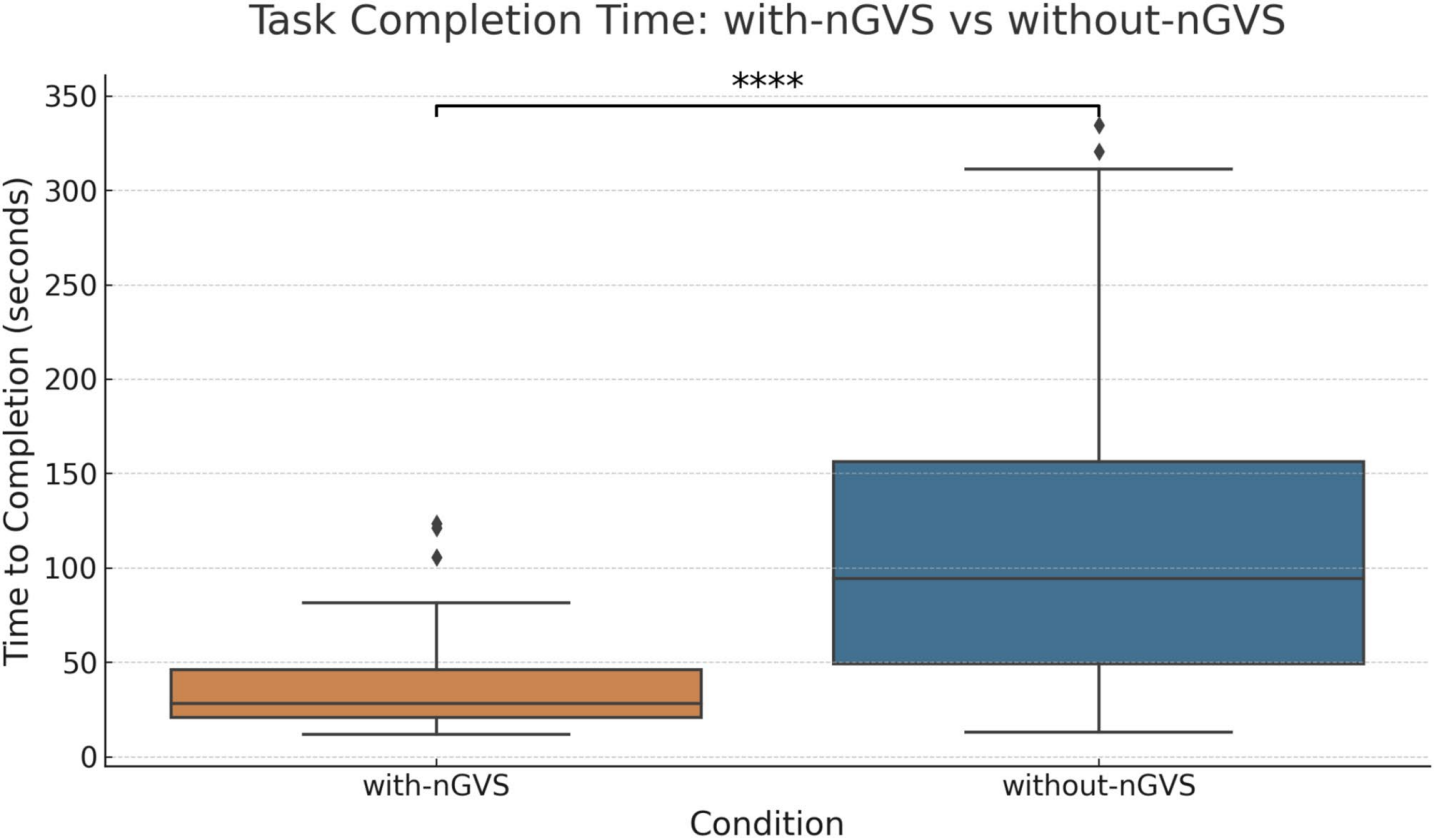
Boxplot illustrating the time to completion for a navigation task under with-nGVS and without-nGVS conditions. The plot clearly shows that the time taken in the with-nGVS condition is notably shorter.

The metric time to completion (TTC) represents the time taken by a participant to complete the multi-stage navigation task. By analyzing this metric, we can gauge performance, i.e., shorter TTC implies faster task time, suggesting better performance. It is important to note that the task was open-ended, with no predetermined time limit for completion. Figure 3 shows a comparison of the time taken to complete a navigation task in both conditions. We compared the (TTC) between the two study conditions (with-nGVS and without-nGVS) using The Mann-Whitney U test ($U = {983}$, $p < 0.0001$). The low p-value indicates the presence of a statistically significant distinction in TTC between the with-nGVS and without-nGVS conditions.

### Gaming experience and VR task performance

We explored the influence of gaming experience on performance in the study task, specifically examining the metrics of time to completion (TTC) and path length (PL). Participants were separated into two groups in each condition: gamers and non-gamers based on their self-reported data collected in the pre-study questionnaire.

#### Overall performance

The statistical analysis did not reveal significant differences between gamers and non-gamers in terms of TTC ($U = {2776},\, p = {.430}$) or PL ($U = {2601}, \,p = {.160}$). These outcomes indicate that video gaming experience did not have a significant impact on spatial cognition in VR in this study.

#### Condition-specific performance

Within the with-nGVS condition, there were no significant differences found between gamers and non-gamers for both TTC ($U = {870}, \,p = {.426}$) and PL ($U = {779}, \,p = {.938}$). Similarly, the without-nGVS condition also showed no significant differences in TTC ($U = {619}, \,p = {.480}$) and PL ($U = {599}, \,p = {.361}$).

These findings indicate that gaming experience did not have a significant impact on the primary performance metrics in our experiment, regardless of the condition. However, given the nature of this study and the small sample size, further investigation may be warranted to confirm these results.

### SBSOD baseline measure

We used SBSOD^27^ as a baseline measure to ascertain participant self-perception of spatial navigation and orientation abilities. The scoring of SBSOD is systematic, with responses to its items being aggregated to produce a composite score. Higher scores therefore indicate a stronger self-perceived sense of direction, while lower scores suggest the opposite. This baseline, thus, provides a reasonable framework to contextualize and interpret navigational performance in our experiment. Average score for participants in the with-nGVS condition was 4.51 (max. 7) and for without-nGVS condition was 4.61 (max. 7). Average SBSOD score for males was 4.50 and females was 4.42 indicating a nearly analogous self perceived baseline.

Navigational performance, assessed through TTC and PL, was analyzed in relation to SBSOD scores using Spearman’s rank correlation ($\rho$). Spearman’s rank correlation coefficient ($\rho$) was chosen due to its non-parametric nature, making it suitable for the non-normally distributed data observed in our study. It is particularly effective for analyzing ordinal data, like the SBSOD scores, and is robust against outliers. Additionally, Spearman’s correlation is capable of identifying monotonic relationships, whether linear or nonlinear, which is advantageous when the precise nature of the relationship between variables is not predetermined. These attributes made Spearman’s correlation appropriate for examining the association between participants’ self-reported sense of direction and their performance in the navigation task in our VR environment.

Analysis revealed no statistically significant relationship for TTC ($\rho = {0.125}$, $p = {0.497}$) and PL ($\rho = {0.112}$, $p = {0.5401}$), suggesting higher SBSOD scores did not correlate with better performance for time and path length. Condition-specific analyses in the with-nGVS condition showed non-significant but moderately positive correlations for both TTC ($\rho = {0.428}$, $p = {0.097}$) and PL ($\rho = {0.269}$, $p = {0.313}$). In contrast, the without-nGVS condition exhibited non-significant moderately negative correlation for $T_c$ ($\rho = {0.133}$, $p = {0.624}$) and no correlation for $P_l$ ($\rho = {-0.035}$, $p = {0.896}$), indicating that the absence of nGVS may allow for a clearer manifestation of the relationship between SBSOD scores and navigational performance.

### Frequency analysis

**Fig. 4:**
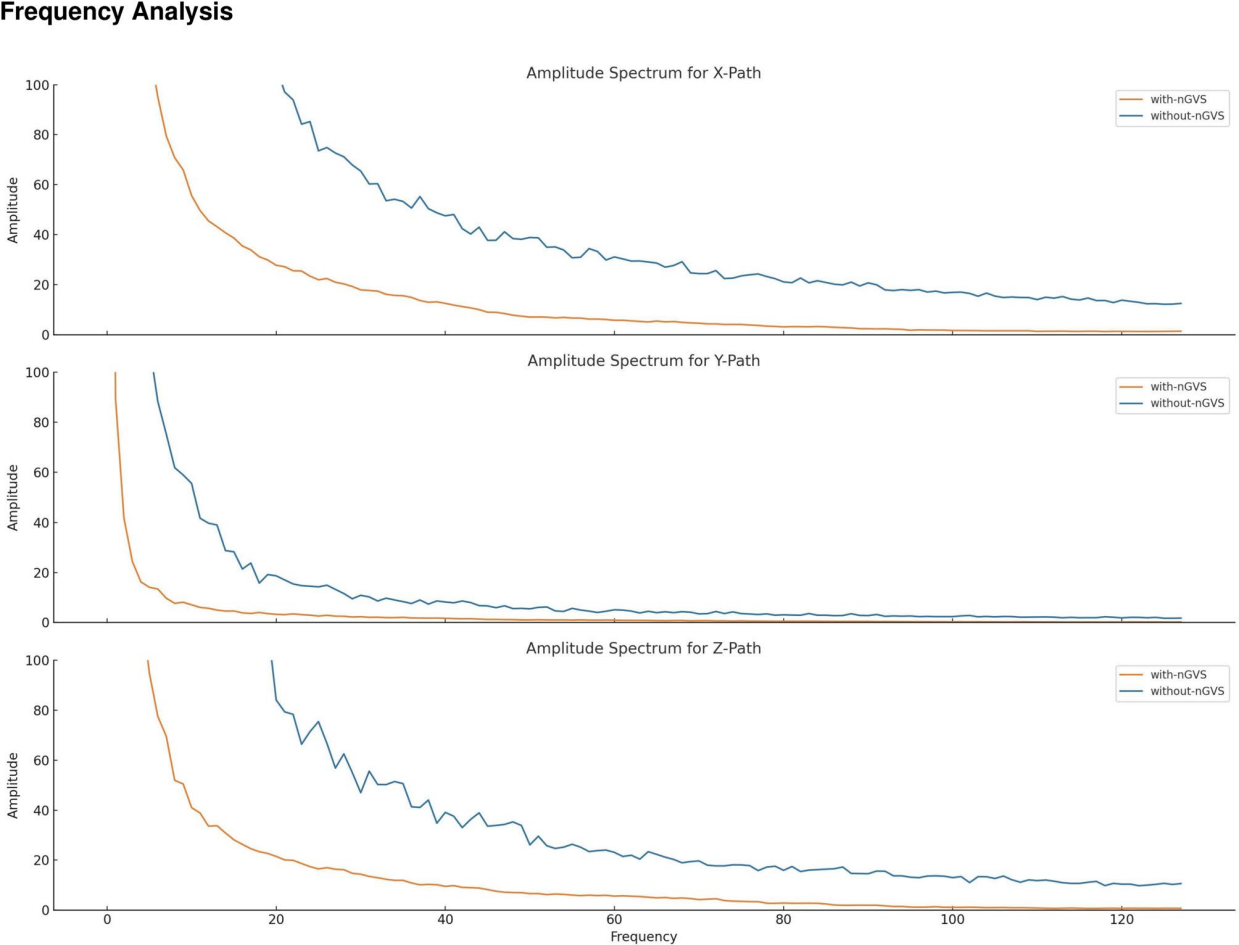
Cumulative FFT plots for X, Y, and Z dimensions, comparing with-nGVS and without-nGVS conditions. These plots visually represent the frequency components of movement in each spatial dimension, revealing distinct patterns between the two conditions.

Using Fast Fourier Transform (FFT), we transformed path data from the time domain to the frequency domain, to allow us to identify dominant frequencies in participant movements (Fig. 4). This could potentially reveal if there are any periodic or rhythmic movement patterns present in the paths. Before applying FFT, we needed to ensure the data is evenly spaced in time. Given that we know the total time taken for each task, we follow a standard sampling rate of 6Hz, interpolating the paths to get evenly spaced data points. Next, we transform the path data into the frequency domain and analyze the dominant frequencies in the transformed data. Based on the FFT analysis performed for movement data across subtasks 1 to 5 for both the with-nGVS and without-nGVS conditions, we make the following observations:

1. *Distinct dominant frequencies* Both conditions, with- and without-nGVS, presented distinct dominant frequencies for each subtask. These frequencies represent the most common periodic movement patterns observed in the data.
2. *Variability in dominant frequencies* In several subtasks, the dominant frequency for the without-nGVS condition was slightly higher than that for the with-nGVS condition. A higher frequency suggests more frequent changes in direction or more oscillatory movement behavior. Conversely, a lower frequency indicates smoother, more consistent paths.
3. *Amplitude differences* The amplitude of the dominant frequencies also showed variability between the two conditions. The amplitude indicates the prominence or strength of the movement patterns corresponding to those frequencies. Differences in amplitude suggest that one condition may have had more pronounced movement patterns than the other for certain subtasks.

From these observations, we infer the following:

- *Movement patterns* Participant movement patterns were not consistent across the two conditions. The presence of distinct dominant frequencies in the FFT analysis suggests that the two conditions influenced the paths differently.
- *Consistency in movement* The with-nGVS condition might have led to more consistent or smoother paths in certain tasks, given the slightly lower dominant frequencies compared to the without-nGVS condition. This could be an indication of the impact of the vestibular stimulation on participant’s sense of balance or spatial orientation.
- *Prominence of movement patterns* Differences in amplitude for dominant frequencies between the two conditions imply that certain movement patterns were more pronounced or noticeable in one condition compared to the other.

It is worth noting that while the FFT analysis provides insights into the frequency components of the movement data, it does not offer a full understanding of participant behavior or the reasons behind these patterns or the influence of the chosen VR navigation technique. Further investigation is needed to draw more definitive conclusions from these observations.

### Participant feedback

Open-ended feedback from the participants revealed insightful perspectives on their experience and perception of the study. Notably, a significant portion of those in the with-nGVS condition (15 out of 16) reported experiencing no perceptible stimulation from the nGVS nor any motion sickness. Conversely, 5 of the 16 participants in the without-nGVS condition reported experiencing some motion sickness and nausea.

“Looking for that fire had me seriously frustrated. And by the time I hit the fifth task? I was feeling all kinds of motion sick” [P7, without-nGVS]. Recalling locations was challenging for most in the without-nGVS condition, with participants only having a vague idea of the fire locations. “I was literally testing my luck, wandering every nook and cranny hoping to spot that fire. Ended up trekking across the map more than once” [P1, without-nGVS]. Another participant described their “zig-zag” approach to traversing the map which underscores the trial-and-error method adopted by them to locate the fires.

In our post-experiment feedback, the median score for motion sickness on the 7-point Likert scale was 3.5. Five participants in the without-nGVS group reported motion sickness post-experiment compared to only one participant in the with-nGVS condition, echoing previous research that highlights GVS’s potential in reducing VR-induced motion sickness^17^.

This feedback results suggests that nGVS might have had a positive impact on reducing motion sickness in the VR environment, and therefore, positively influenced navigation strategies. However, given the small sample size and nature of this study, further research is needed to confirm the pathway of these observations and explore their implications.

## Discussion

We explored the impact of noisy GVS on spatial navigation for an object-finding task in VR. In our between-subjects design, 32 participants were split into two groups of with-nGVS and without-nGVS. They performed a navigation task that consisted of five distinct subtasks or stages, each of which required participants to navigate to the location of one of the five fires within the VR scene. While the study is preliminary, the results indicate significant difference in performance between the with-nGVS and without-nGVS conditions.

The MWU showed a decrease in effect sizes for subtasks 4 and 5, as mentioned in Sect. 2.1. This suggests a ceiling effect in task difficulty. It seems that after identifying several target fires, participants may have employed heuristic strategies to locate the others, such as an elimination process, potentially masking the true efficacy of nGVS in enhancing spatial cognition and memory. Furthermore, the relative simplicity and small size of the virtual environment may have allowed participants to acquire substantial spatial understanding within a short period of navigation. This is further supported by the fact that the majority of participants reported high self-ratings on the SBSOD scale.

It is important to note that our study design presents several key distinctions from prior research. Our use of a VR environment offers a more ecologically valid approach compared to screen-based experiments. VR’s egocentric perspective and multimodal sensory cues, including visuals, spatial sound, and proprioception, have been shown to enhance spatial learning, memory, and navigation performance^34–36^. This design choice allows for a closer alignment with real-world navigation scenarios (see review^37^).

For nGVS stimulation used in our experiment, the signal amplitude was below perceptual threshold for each participant, as indicated by them during GVS calibration. Our between-subjects design meant that participants were either in the with-nGVS or the without-GVS condition, but not in both, preventing any direct comparison of conditions or remnant effects of vestibular stimulation.

Our study applies nGVS stimulation during both encoding and retrieval phases of the spatial memory task. This comprehensive approach offers a more complete understanding of nGVS effects on spatial memory compared to studies focusing solely on the encoding phase^38,39^. Additionally, our data analysis techniques, such as Fast Fourier Transform (FFT), provide novel insights into participants’ movement patterns, offering a deeper understanding of the effects of nGVS on spatial navigation^40,41^.

Our analysis revealed no significant relationship between SBSOD scores and performance metrics of TTC and PL. While the with-nGVS condition exhibited non-significant but moderately positive correlations, and the without-nGVS condition showed mixed results with a moderately negative correlation for TTC and no correlation for PL, none of these relationships reached statistical significance. This absence of clear correlation between self-reported spatial abilities and task performance might be attributed to several factors: the limited sample size, the specific demands of our object-finding task, or the constrained nature of the VR environment which provided fewer environmental cues compared to real-world navigation. Moreover, the potential influence of nGVS on vestibular input might modulate how individuals employ their spatial abilities in virtual environments, suggesting an avenue for future investigation with larger sample sizes.

Our findings also did not reveal significant performance differences between gamers and non-gamers, as prior work suggests is the case for game players^42^. Their findings suggest that this applies only to people who regularly play games with a navigational component as they inadvertently hone their spatial orientation and navigation capabilities with frequent gameplay. In our study, this outcome may be attributed to the prolonged encoding phase^43^ and the small size of the virtual environment vital for the consolidation of spatial memory, potentially leveling the playing field between the two groups. While we asked participants about their gaming experience, we did not separate video gaming from VR gaming. It is likely that those with high VR gaming experience would perform differently in a VR study than those without and would be worth investigating in future work.

In our post-experiment feedback, the median score for motion sickness on the 7-point Likert was 3.5. Four participants in the without-nGVS group reported motion sickness post-experiment compared to only one participant in the with-nGVS condition, echoing previous research that highlights GVS’s potential in reducing VR-induced motion sickness^17^. This outcome introduces a challenge in interpreting the results, as the improved navigation performance could stem from reduced VR-induced motion sickness rather than enhanced navigation skills directly caused by nGVS. The research dilemma lies in the fact that factors mitigating motion sickness, such as minimizing visual-vestibular conflict or applying GVS, may inherently boost navigation performance. This overlap makes it difficult to experimentally determine whether the observed improvements are primarily due to decreased motion sickness or the direct effects of these interventions on spatial cognition. While our results demonstrate performance improvements with nGVS, we acknowledge the inherent challenge in fully disentangling the direct effects on spatial cognition from indirect effects via motion sickness reduction. Prior work indicates that GVS modulates hippocampal^20,21^, whereas its motion sickness benefits operate through the nucleus tractus solitarius^33^, suggesting independent mechanisms. Future investigations could incorporate physiological measurements of motion sickness symptoms alongside spatial performance metrics to better isolate these mechanisms, though the significant performance differences observed likely reflect a combination of beneficial effects on the vestibular system. 

A substantial body of cognitive and neurophysiological studies has consistently demonstrated inherent differences in spatial cognition and navigation abilities between males and females, particularly in virtual environments, independent of nGVS use. These differences add another layer of complexity to interpreting the effects of nGVS on spatial navigation performance^44–46^. Our study found no significant gender-based disparities in nGVS-related performance, potentially reflecting our study’s small sample size. This also underscores the necessity to consider multifaceted factors, such as socio-cultural influences, upbringing, and experiential learning, that might contribute to an individual’s spatial cognition and navigation abilities.

Observing the trajectories of participants (without-nGVS condition) revealed varied navigation strategies for fire discovery tasks; like the zig-zag strategy mentioned by participants. Notably, some participants also exhaustively searched every potential location on the map. Given the map’s small scale, this approach did not present significant challenges.

On average, participants completed individual navigation subtasks in approximately 36 seconds under the nGVS condition compared to 113 seconds in the without-nGVS condition, with median completion times of 28 seconds and 94 seconds respectively. The total task completion time across all five subtasks averaged around 179 seconds (about 3 minutes) for the nGVS group and 567 seconds (about 9.5 minutes) for the without-nGVS group

Future studies could benefit from incorporating a sham stimulation condition that provides electrical stimulation to the skin without reaching the vestibular nerve. Such a control would help further distinguish between the specific effects of vestibular stimulation and general effects of electrical stimulation, potentially strengthening the evidence for vestibular-specific effects on spatial navigation.

Lastly, it is worth highlighting our technical innovation in this field. Our custom-built, lightweight, wearable GVS device represents a significant advance over commercially available systems, offering new possibilities for controlled experimentation in spatial memory research. The device’s mobility and ease of use in VR environments opens up new avenues for future investigations in spatial cognition and nGVS research.

Our VR environment was designed to minimize motion sickness-inducing features, such as rapid camera movements, fast-moving objects, and unstable visual references. Participants also reported low susceptibility to motion sickness, which further reduced the likelihood of cybersickness significantly affecting the study. As a result, administering the Simulator Sickness Questionnaire (SSQ) was deemed unnecessary. However, the observed improvement in spatial memory in the with-GVS condition compared to the without-GVS condition introduces a potential confound: GVS is known to reduce motion sickness^17^, which might indirectly enhance spatial memory by alleviating the cognitive and physiological burdens associated with discomfort. This raises the possibility that the observed improvement could be due to reduced motion sickness rather than a direct vestibular enhancement. Although the VR design minimized motion sickness-inducing elements and our results indicate direct impact of GVS on spatial memory, future studies could address the confound by explicitly measuring motion sickness using the SSQ and analyzing its relationship with spatial memory performance. If motion sickness is similarly low across conditions but spatial memory still improves significantly with GVS, this would strengthen the case for a direct vestibular enhancement.

## Materials and methods

### GVS hardware design

**Fig. 5:**
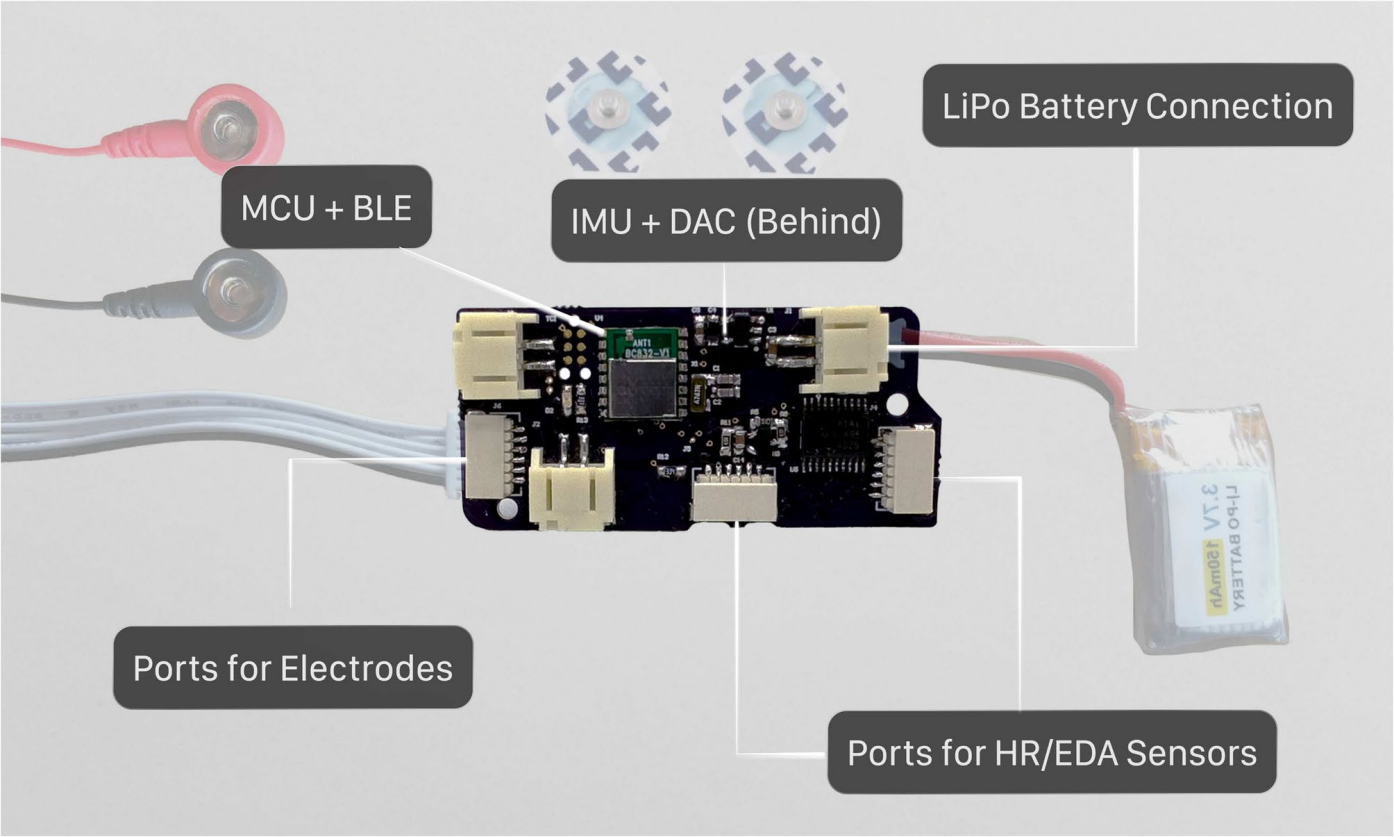
Our custom made GVS (Galvanic Vestibular Stimulation) device. It uses BLE (Bluetooth Low Energy) to interface with an iOS app. PCB, LiPo battery, sensor ports, and the electrode connectors are shown in the figure.

For this work, we used a custom built lightweight wearable GVS device (Fig. 5). While participants in this study remained seated due to space constraints, our portable design offers advantages including improved comfort, minimal interference with VR equipment, and crucially, enables future research investigating GVS effects during natural movement and mobile applications.This is in contrast with existing GVS devices available for purchase that are usually heavy and designed for seated desktop use^47^. We use controlled voltage sources with individual current sensors to create a software-controlled current source and produces a biphasic 15V output. The device’s main printed circuit board (PCB) is embedded with a system-on-chip module, responsible for sensing, actuating, and transmitting data via Bluetooth Low Energy (BLE). It incorporates an on-board boost converter, eliminating the need for 9V batteries, and is equipped with a 3-channel current driver and a corresponding sensor for each channel. To accommodate future enhancements, a 6-axis inertial measurement unit (IMU), a low-power digital-to-analog converter (DAC), and two H-bridge drivers for actuators have been integrated. The circuit is powered by a single-cell 150mAH LiPo (lithium polymer) rechargeable battery, offering approximately 8 hours of continuous usage.

The GVS device’s operation is managed through an iOS application, designed to allow precise control over the initiation of GVS stimulus and the acquisition of sensor data. This custom interface allows real-time adjustments to stimulus parameters (magnitude, frequency, mode etc.) and also serves as a critical conduit for ensuring user safety.

#### Safety considerations

Safety was of paramount importance and we designed our device to ensure the peak current drive capability is restricted to 1.46 mA, significantly below the thresholds of comparable systems and commercial TENS units^47,48^. The device incorporates passive resistors for current limitation, safeguarding against potential overcurrent scenarios. Thin film resistors, when subjected to overstress, exhibit a tendency to increase their resistance, leading to an open circuit^49^. This inherent safety mechanism acts as an additional layer of protection. Our device was validated by an external evaluator (a professional electrical engineer) for robustness and reliability of the device’s safety features, confirming its adherence to established safety norms and standards and to help meet IRB safety requirements.

As a judicious precaution, a direct current (DC) ammeter measurement between each electrode is conducted prior to beginning the study with each participant to ensure current values are in the expected range.

### Participants

Our study included 32 volunteers (16M, age range: 19–28 years, mean age: 23.25 ± 3.45 years; 16F, age range: 19–48 years, mean age: 26.94 ± 6.73 years). Gaming habits, pivotal due to their potential influence on spatial navigation in immersive settings^50^, exhibited variation among participants. Out of the total, 12 identified as regular digital gamers (2+ hours of gaming daily), while 20 reported no regular gaming activity. Both groups (i.e., with-nGVS and without-nGVS) presented an equitable distribution: 6 gamers and 10 non-gamers in each. Nobody specified playing VR games. This deliberate composition served to mitigate any undue biases that might stem from gaming familiarity.

Sensitivity to motion sickness was assessed using a subjective pre-study questionnaire, revealing a median score of 3.0 on a 7-point scale, where 1 indicated minimal sensitivity and 7 indicated extreme sensitivity. This low score suggested that the participants had generally low susceptibility to cybersickness.

An explicit set of exclusion criteria were defined for the study to meet IRB requirements. Participants with susceptibility to motion sickness, vestibular disorders, neurological disorders or diagnoses (like seizure disorder, stroke, balance disorder, or gait disorder), and color blindness were requested not to volunteer. The study employed stimulation of the vestibular system with electrical current, which could potentially interfere with medical devices. Thus, as a precautionary measure, participants with pacemakers, implanted stimulators, non-removable hearing aids, head and neck tattoos, non-removable piercings, migraines, non-electrical implants like metal pins or joints, hearing impairments, tinnitus, or specific low vision conditions unsupported by consumer VR devices, were also excluded.

Prospective participants were informed of these criteria in the recruitment email, and potential volunteers were pre-screened using an online questionnaire. Those meeting any exclusion criteria were subsequently informed of their non-selection.

The study received approval after a full board review from our local Committee on the Use of Humans as Experimental Subjects (UCSB Human Subjects Committee protocol # 19-24-0243). Prior to the experiment, all participants were briefed on the study’s objectives and protocols, following which they provided informed consent.

### Apparatus

We used the Meta Quest 1 VR headset (HMD) wirelessly connected (casting) to a MacBook Pro. The experiment was conducted in a sound-proof, quiet room (4 m × 4 m in size). Disposable MyoWare hydrogel electrodes (30 × 20 mm) were placed on the user’s mastoid process behind each ear to connect the GVS device.

For the VR application, we created a virtual environment using Unity and the Oculus integration package which provides development support for Oculus VR devices. We used freely available 3D assets from the Unity Asset Store^51^ to build the VR environment. The virtual setting resembled a sea port, featuring proximal elements like boats, towers, and gazebos, surrounded by the ocean. We added distal cues such as stationary clouds and the sun to provide a realistic spatial context, which in the real world provide stable reference points for orientation and navigation over larger areas. To more closely reflect a real world setting, we incorporated a stereo channel soundscape of a seaport (complete with the sounds of crashing waves, ambient noises such as bells and birds, and other harbor sounds). This auditory environment was delivered to participants through the two built-in speakers of the Quest HMD, allowing them to audibly immerse themselves in a setting that closely mirrors the real world.

**Fig. 6:**
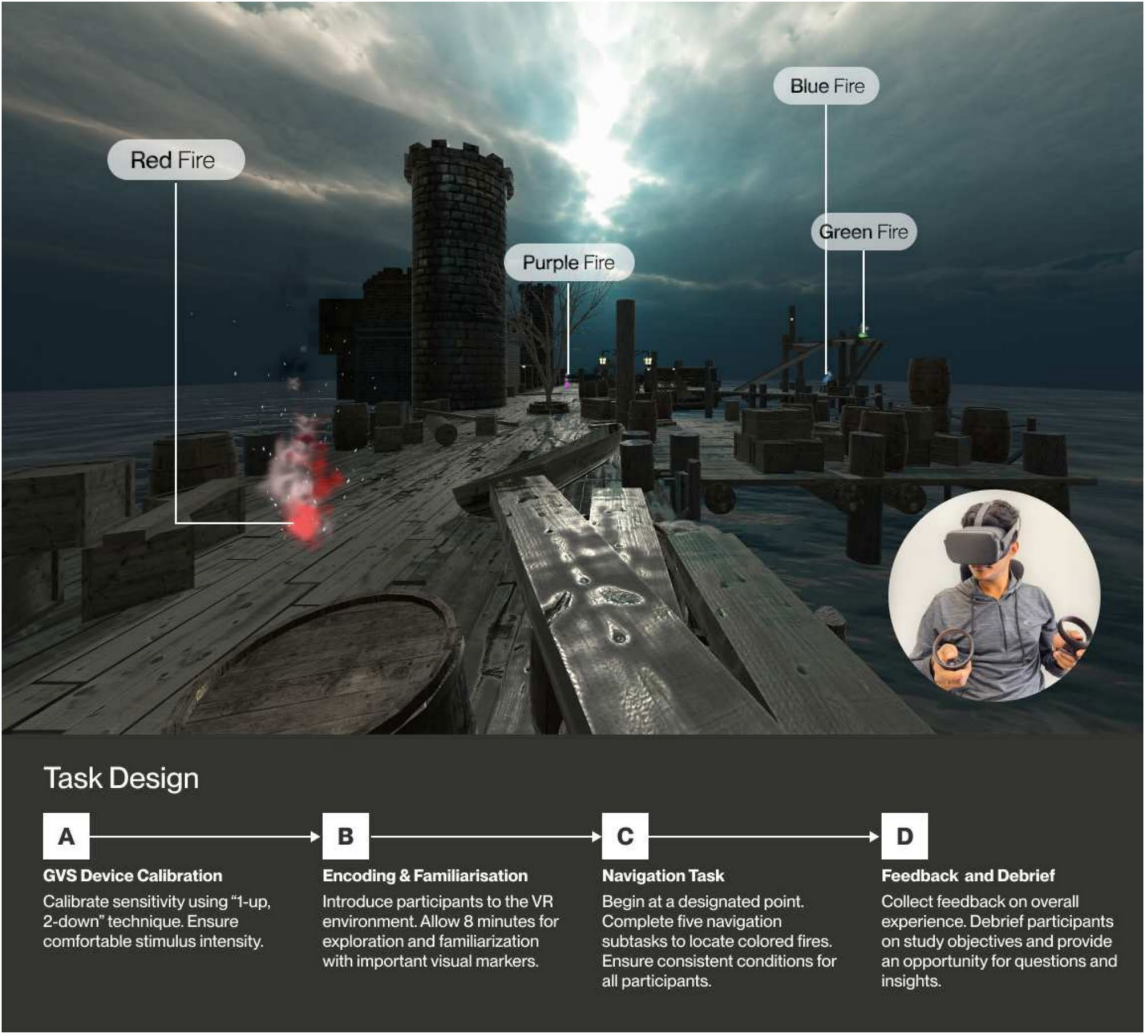
A participant’s first person point of view in the VR environment with study task objects or fires visible or their locations labeled; (Inset) Participant performing the experiment with Meta Quest 1 Headset. Figure created using Figma^52^.

### Experiment design

Our study’s goal was to explore the effects of nGVS on spatial navigation for an object-finding task in VR. In our between-subjects design, participants were split into two groups: with-nGVS and without-nGVS. This decision was threefold: firstly, to mitigate the learning effect, secondly, to account for the lingering effects of vestibular stimulation, which could potentially skew results if both conditions were experienced by the same participant^53^, and lastly, to support data collection in a way that did not rely on participants returning for follow-up sessions.

Our study task is a modified version of the Morris water maze task^54^, considered the gold standard test of animal navigation in laboratory settings. We introduce a multi-stage navigation task, which represents a significant departure from typical screen-based spatial cognition studies involving humans. Traditional experiments, as described by Hilliard et al.^32^, often rely on simple object placement and recall tasks conducted on an open plane. Our approach, however, involves a more complex and realistic navigational scenario, better reflecting real-world spatial cognition demands. The sequential navigation task requires participants to locate items one after another. By avoiding the repetitive start-reset cycle, where participants always begin at a fixed starting point and orientation as seen in prior work^32^, our design mirrors more realistic navigation scenarios.

Specifically, our multi-stage navigational task comprises five subtasks or stages, each centered around finding one of five distinctly colored fires (red, blue, green, yellow, and purple) positioned at various locations in the virtual environment (Fig. 6). The selection of five objects to locate is grounded in prior research indicating an optimal balance between cognitive load and task complexity, allowing for comprehensive assessment of spatial memory without overwhelming participants^55^. Our methodology differs significantly from that of Hilliard et al.^32^. While their screen-based spatial memory experiment placed various objects on a flat 3D plane, we use a VR environment with a more complex spatial arrangement. In our study, we used a single type of object-fires-but varied their colors and positions. Importantly, these fires were not all placed at ground level; some were positioned at different heights within the VR scene. This 3D arrangement introduces additional spatial complexity compared to the layouts used in traditional screen-based experiments. To prevent participants from accidentally discovering the fires during navigation, we strategically placed all five fires at distinct, widely separated locations within the virtual environment. The placement strategy ensured that participants would need to intentionally explore the environment to locate each fire, rather than stumbling upon them.

Participants underwent a calibration process for the GVS device (see Section on GVS Calibration). After calibration, participants familiarized themselves with the VR controls^56^, followed by an 8-minute encoding phase during which they were encouraged to actively explore the virtual environment with the objective of locating the five fires. The choice of an 8-minute duration for the encoding phase is supported by research indicating that prolonged exposure significantly enhances spatial memory consolidation and retention^43^. The encoding phase is aimed at maximizing environmental interaction, thereby boosting the efficiency of memory encoding and overall spatial performance for all participants.

By separating VR control familiarization from the encoding phase, our intent was to eliminate potential confounding factors such as unfamiliarity with the interface and cognitive overload. This deliberate design choice facilitated a focused encoding of spatial information, devoid of the need to simultaneously learn control mechanics, to promote a more effective learning environment.

The study task began after the encoding phase and involved navigating to all the fires, one by one. To start, a participant was positioned in the western end of the VR environment, with all fires removed, and presented with text-based instructions in VR to find their way to the first fire. After successful navigation to the correct location of the first fire, the participant was re-positioned in the scene to find their way to the next fire and so on. Every new starting position and orientation was chosen so as to not afford a direct line of sight to either the previously encountered or the upcoming fire.

Each participant performed the task once with participants in the with-nGVS condition stimulated during the entire duration of the encoding phase and the task. The order of fires presented to each participant was maintained to allow a fair comparison between subjects, i.e., each participant went to the red fire first followed by blue, green, yellow and purple fires. The locations of the fires was not changed between participants, nor were the participant starting points.

Participants performed the study task seated and navigated the VR environment using joystick controls. While teleportation is the most widely used navigation strategy in VR, especially when physical space is limited^57^, we implemented joystick-controlled navigation in our study. This decision was made because teleportation allows users to instantly move between points in the virtual environment, potentially skipping over significant portions of the space. Such instant movement could interfere with participant ability to form a comprehensive mental map of the environment. Joystick control, on the other hand, requires users to traverse the entire space, promoting a more complete and continuous spatial understanding of the virtual environment. Compared to other artificial interaction types like head-directed navigation, joystick-controlled navigation also offers better spatial orientation, ease in changing direction, and more accurate movement^58,59^. Upon the conclusion of the study task, participants were asked to reflect on their experience and provide open-ended feedback.

### GVS calibration

Calibrating the GVS device for each individual was necessary to establish the device’s performance range according to individual sensitivity to vestibular stimulation^17,53^. This pre-process helped us identify the effective amplitude range for each individual, ensuring the safety and comfort of the vestibular stimulation during the study. To calibrate, we asked each participant in the with-nGVS condition to walk in a straight line, with their eyes closed and head tilted downward. Subsequently, the Bluetooth-enabled GVS device administered stimulation to the participant’s mastoids using EMG (H124SG) electrodes. The anodal-left current was initiated at 0.5 mA and progressively increased in increments of 0.2 mA until a leftward sway of approximately 20 degrees was observed in the participants gait^53^.

The effective amplitude corresponding to the sway was used as the calibrated range of nGVS for the experiment. The “1 - up, 2 - down” adaptive staircase technique was employed to ascertain the absolute detection threshold. Participants were asked to rate their sensations experienced during vestibular stimulation on a 5-point scale^60^, to help establish their effective range. The scale is as follows:

1. No sensation
2. Barely perceptible tingling
3. Moderate tingling, bordering on discomfort
4. Pronounced discomfort
5. Intense discomfort or pain

We interpreted a rating exceeding 2 to indicate discomfort at the mastoid, necessitating a downward adjustment in the stimulus intensity to ensure comfort.

### Procedure

The study started with participants arriving at the designated location, where they received an overview of the study’s procedures and goals. The study was approved by the UCSB Human Subjects Committee, which serves as the IRB (study protocol # 19-24-0243) to review research applications involving human subjects. All participants provided informed consent before proceeding with the study. All research was performed in accordance with relevant guidelines/regulations. See Fig. 6 for study virtual environment and procedure.

- *Consent and preliminary information* Before any interaction, participants were presented with a consent form, outlining the study’s objectives, the nature of the study task, and potential risks. Participants were provided an information sheet about the GVS device, and the researcher answered any questions or concerns about vestibular stimulation, before beginning the study.
- *Santa barbara sense of direction scale (SBSOD)* As an initial step, participants completed the SBSOD questionnaire^61^. This self-report measure was used to gauge their inherent spatial navigation abilities.
- *GVS device calibration* The GVS device was calibrated to each participant’s sensitivity to establish the effective amplitude range using the “1-up, 2-down” adaptive staircase technique^62^. Participants reported sensations using a 5-point GVS sensation scale, and we ensured the stimulus intensity remained within comfortable limits.To control for potential sensory and tactile cues from wearing the device, participants in both groups wore the GVS equipment, with those in the without-GVS condition wearing the device but receiving no electrical stimulation. Participants were not informed which condition they were assigned to.
- *Encoding and familiarization phase* Participants were subsequently introduced to the virtual environment, equipped with the Meta Quest 1 headset and controllers. They were guided through the functionality of the joystick controls in the virtual space. Following this orientation, they were given 8 minutes to navigate through the environment, with a specific emphasis on becoming familiar with essential visual markers, to identify the locations of the colored fires. During this period, any questions or uncertainties they encountered regarding the virtual environment were addressed and clarified.
- *Navigation task* After familiarizing themselves with the virtual environment, participants were teleported to a designated starting point to begin the navigation task for the study. This multi-stage task consisted of five distinct subtasks or stages, each of which required participants to navigate to the location of one of the five fires within the VR scene. At the starting point, they were given a text prompt in VR that specified their immediate navigation target, such as, “Navigate to the location of the green fire.” A subtask was considered successfully completed once a participant accurately navigated to the bounding box coordinates corresponding to the specified fire’s location, with the bounding box designed to match the fire’s dimensions on a 1:1 scale. Participants were not given any time constraints and could take as long as needed to complete each subtask. Upon completing a subtask, participants were moved to a new starting point to navigate to the next colored fire. Each subtask required participants to navigate to a different colored fire. All participants were placed at the same starting location and orientation to maintain a controlled and uniform experiment setup. This consistency ensured that all participants faced identical conditions for each subtask, facilitating a fair comparison of their navigation abilities.
- *Feedback collection* Upon completion of the navigation task, participants were asked to share any feedback on their overall experience. Additionally, they were asked to evaluate any motion sickness experienced during the study by responding to the same motion sickness-related question as in the pre-study questionnaire, using a 7-point Likert scale.
- *Conclusion and debrief* Finally, participants were debriefed about the study’s broader objectives and implications. They were also given an opportunity to ask questions or share additional insights about their experience.

## Data Availability

The experimental datasets used and/or generated during this study are available from the corresponding authors upon reasonable request.
